# Nostalgia Proneness and the Collective Self

**DOI:** 10.3389/fpsyg.2020.570621

**Published:** 2020-10-26

**Authors:** Georgios Abakoumkin, Tim Wildschut, Constantine Sedikides

**Affiliations:** ^1^ Laboratory of Psychology, Department of Early Childhood Education, University of Thessaly, Volos, Greece; ^2^ Centre for Research on Self and Identity, School of Psychology, University of Southampton, Southampton, United Kingdom

**Keywords:** nostalgia proneness, relational collectivism, group collectivism, ingroup identification, collective self

## Abstract

In two studies, we examined the association between nostalgia proneness (i.e., trait-level nostalgia) and importance of the collective self. In Study 1, we tested and supported the hypothesis that nostalgia proneness is positively correlated with relational collectivism, which entails an emphasis on one’s connections with close others and small social networks. In Study 2, we demonstrated that nostalgia proneness is also positively correlated with group collectivism, which emphasizes one’s membership in more abstract, larger social groups or categories, and was reflected in increased identification with a national ingroup. These findings offer insight into the nature of nostalgia proneness—a consequential and stable personality trait.

## Introduction

Nostalgia, “a sentimental longing … for the past” (Pearsall, 1998, p. 1266), is an ambivalent, albeit predominantly positive, and social emotion (Hepper et al., 2012a; Sedikides and Wildschut, 2016; for historical overviews, see Batcho, 2013; De Diego and Ots, 2014). When people nostalgize, they feel connected with others in a way that enhances their perceptions of belongingness and acceptance (Sedikides et al., 2015, 2016; Abakoumkin et al., 2019). In nostalgic reverie, “the mind is ‘peopled’” (Hertz, 1990, p. 195). Mental representations of social bonds (e.g., family, friends, and partners) form the building blocks of nostalgic memories; important figures from one’s past are brought to life and become part of one’s present (Davis, 1979; Batcho, 1998; Abeyta et al., 2015; Wildschut et al., 2018). As a result, nostalgia makes one feel loved, supported, and efficacious in social relations (Zhou et al., 2008; Wildschut et al., 2010). However, most of the research on the sociality function of nostalgia has been concerned with momentary or transient (i.e., state-level) nostalgia. In the present investigation, we focus on the role of trait-level nostalgia (i.e., nostalgia proneness; Sedikides et al., 2004; Wildschut and Sedikides, in press).

The extant literature indicates that nostalgia proneness is positively related with various indices of sociality. High-nostalgia (compared to low-nostalgia) individuals value social inclusion. In an early study (Batcho, 1998), participants first completed the Nostalgia Inventory (NI; Batcho, 1995), which assesses nostalgia proneness across different aspects of everyday life. Specifically, participants rated how much they generally missed each of 20 items from when they were younger (e.g., “someone I loved,” “the way people were,” “my pets”). The average rating was used to classify participants as high nostalgia and low nostalgia by selecting the top and bottom 25% of the sample, respectively. Next, participants rated themselves on 10 aspects of personality, including the extent to which they preferred activities with people rather than alone. High-nostalgia (compared to low-nostalgia) participants reported a stronger preference to be with other people rather than alone.

More recently, Juhl et al. (2020) showed the positive associations of nostalgia proneness with three core features of human sociality: empathy (Eisenberg and Strayer, 1987), attachment security (Mikulincer and Shaver, 2011), and prosocial or charitable behavior (Batson, 2011). Across five studies, nostalgia proneness was consistently associated with greater affective empathy (assessed with various measures; e.g., “Seeing people cry upsets me”; Mehrabian and Epstein, 1972), including among young children. Moreover, the link between nostalgia proneness and affective empathy held when controlling for domain-level (Big Five) personality traits and did not vary significantly as a function of age or gender. Juhl et al. further showed that high-nostalgia (compared to low-nostalgia) individuals evinced greater attachment security (assessed with the Security subscale of the Attachment Style Questionnaire; e.g., “I trust other people and I like it when other people can rely on me”; Hofstra et al., 2005). Attachment security, in turn, mediated the relation between nostalgia proneness and empathy. High-nostalgia (compared to low-nostalgia) individuals were more likely even to donate money to charity when given the opportunity to do so, and this relation was mediated serially by attachment security and affective empathy.

The distinctive sociality of nostalgia proneness is brought into sharp relief when nostalgia is contrasted with two other modes of thinking about one’s past: rumination (Rusting and Nolen-Hoeksema, 1998) and counterfactual thinking (Epstude and Roese, 2008). Cheung et al. (2018) examined how nostalgia, rumination, and counterfactual thinking are similar or different in terms of their relation with seven functions of autobiographical memory (i.e., usages of memory or motives to remember; Harris et al., 2014): intimacy maintenance (attaining symbolic proximity to close but absent others), teach/inform (transmitting insights about oneself or life), self-regard (carrying over effective problem-solving strategies to present action, clarifying one’s identity), bitterness revival (rekindling resentment for having been wronged by others), conversation (enlivening current social exchange), boredom reduction (counteracting tedium), and death preparation (coping with mortality awareness). The uniqueness of nostalgia proneness (compared to rumination and counterfactual thinking) resided in its comparatively strong positive association with intimacy maintenance and weak association with bitterness revival. Nostalgia-prone individuals, then, indicate that they use autobiographical memories to stay connected to loved ones and not to stir up bitterness toward others.

In light of this cumulative evidence, we propose that nostalgia-prone individuals assign greater importance to the collective self. Whereas the individual self refers to those unique aspects of the self-concept that differentiate the person from others and distinguishes him or her within the social context, the collective self refers to those aspects of the self-concept that are shared with other members of the social groups to which one belongs and is based on social bonds to others (Sedikides and Brewer, 2001). Brewer and Chen (2007) distinguished between two forms of self-definition at the collective level, with distinct implications for psychological functioning. Relational collectivism refers to an emphasis on one’s bonds to close others and small social networks. Group collectivism denotes an emphasis on one’s membership in more abstract, larger social groups or categories. In Study 1, we tested the association of nostalgia proneness with relational collectivism (and individualism). In Study 2, we extended this line of inquiry by examining the link between nostalgia proneness and group collectivism, as reflected in (national) ingroup identification. Our overarching objective was to tighten the nomological net around nostalgia proneness and thereby better understand this consequential and stable personality trait (Wildschut and Sedikides, in press).

## Study 1

In Study 1, we used survey data available from the Longitudinal Internet Studies for the Social Sciences (LISS) panel. Participants completed measures of nostalgia proneness, individualism, and relational collectivism. The measures of individualism and collectivism incorporated a further, cross-cutting distinction between a horizontal and vertical orientation (Singelis et al., 1995; Triandis and Gelfand, 1998). A horizontal orientation emphasizes similarities between oneself and others. Thus, horizontal individualism involves self-reliance without a desire to differentiate oneself by achieving high status, and horizontal collectivism emphasizes interdependence with others without submitting oneself to others. A vertical orientation emphasizes hierarchies and differences between oneself and others. Accordingly, vertical individualism involves distinguishing oneself through competition and status acquisition, and vertical collectivism involves subordinating and sacrificing oneself for the sake of one’s social relations. We hypothesized that nostalgia proneness would be positively related to both horizontal and vertical collectivism dimensions (but not to individualism dimensions).

### Method

#### Data Collection

The LISS panel^1^ includes members of the Dutch general public selected based on a true probability sample of Dutch households. Panel members complete studies each month, and their responses can be combined across studies. Data collection is managed by CentERdata in Tilburg, The Netherlands. We assembled the dataset from three LISS studies. “Background Variables” (completed April 2011) contained demographic measures, “Nostalgia—part 1, wave 1” (completed November 2012) contained measures of nostalgia proneness, and “Self-Regulatory Orientation” (completed April 2011) contained measures of collectivism and individualism. Data for the “Background Variables” study are updated monthly, and we used the data collected in the same month as the “Self-Regulatory Orientation” study. The “Nostalgia” study assessed nostalgia proneness at six timepoints. We selected the timepoint (part 1, wave 1) that was closest to the “Self-Regulatory Orientation” study.

#### Participants

Eight hundred sixty-eight participants completed measures of nostalgia proneness, collectivism, and individualism, as well as demographic information (457 women, 411 men; *M*_age_ = 52.13 years, *SD*_age_ = 16.40 years, Range_age_ = 16–91 years).

#### Nostalgia Proneness

Participants in the “Nostalgia—part 1, wave 1” LISS study completed the Southampton Nostalgia Scale (SNS; Barrett et al., 2010) and the NI (Batcho, 1995). For the SNS, they responded to seven items. Three of them measured the extent to which participants found nostalgia valuable, important, or significant (1 = *not at all*, 7 = *very much*), and four measured how frequently participants became nostalgic (1 = *very rarely*, 7 = *very frequently*). We averaged responses to create SNS scores (α = 0.94; *M* = 4.12, *SD* = 1.23). For the NI, participants indicated how nostalgic (1 = *not at all*, 6 = *very much*) they felt about 20 objects (e.g., “someone I loved” and “my childhood toys”). We averaged responses to create NI scores (α = 0.93; *M* = 3.83, *SD* = 1.11). We used two measures of nostalgia proneness for the purpose of convergent validation (Campbell and Fiske, 1959; Wildschut and Sedikides, in press).

#### Individualism and Relational Collectivism

Singelis et al. (1995) developed 32 items to assess horizontal collectivism, vertical collectivism, horizontal individualism, and vertical individualism (eight items for each dimension). Triandis and Gelfand (1998) subsequently adapted 27 of these items to assess the four dimensions. Participants in the “Self-Regulatory Orientation” LISS study completed 8 of these 27 items (2 items for each dimension). These eight items were selected on the basis of their high factor loadings, as reported by Triandis and Gelfand. Participants rated the items (1 = *totally not applicable*, 7 = *totally applicable*). We averaged responses to create indices of horizontal collectivism (“If an acquaintance gets a prize, I would feel proud,” “To me, pleasure is spending time with others”; α = 0.59; *M* = 4.77, *SD* = 1.25), vertical collectivism (“It is my duty to take care of my family, even when I have to sacrifice what I want,” “It is important to me that I respect the decisions made by my groups”; α = 0.63; *M* = 4.81, *SD* = 1.30), horizontal individualism (“I’d rather depend on my own strength than being dependent on others,” “My personal identity, independent of others, is very important to me”; α = 0.70; *M* = 5.28, *SD* = 1.22), and vertical individualism (“Winning is everything,” “Competition is the law of nature”; α = 0.78; *M* = 3.36, *SD* = 1.39). The targets referred to in the collectivism items are not large social groups or categories, but specific interpersonal relationships or networks (e.g., family, friends, and acquaintances). Only one of the four collectivism items referred to groups (“It is important to me that I respect the decisions made by my groups”), and even this item alludes to relatively small social networks (i.e., “*my* groups”) rather than large collectives. For this reason, the present measures tap relational (as opposed to group) collectivism. We acknowledge that the reliability coefficients are lower than desired, particularly for horizontal collectivism and vertical collectivism. This is expected, given that each index comprised only two items. Low reliability attenuates effect size and, hence, reduces statistical power (Cohen, 1988). Fortunately, our sample size (*N* = 868) affords sufficient statistical power (>0.80) to detect even a small effect (*r* = 0.10, two-tailed).

### Results and Discussion

We present zero-order correlations among study variables in Table 1. As hypothesized, both measures of nostalgia proneness (SNS and NI) were significantly and positively correlated with both (relational) collectivism dimensions (horizontal collectivism and vertical collectivism). The nostalgia proneness measures were also positively correlated with both individualism dimensions, albeit less strongly than with the collectivism dimensions.

**Table 1:** Correlations among nostalgia proneness, collectivism, individualism, and demographics in Study 1.

S. No.	Measure	1	2	3	4	5	6	7
1.	Nostalgia proneness (SNS)	--						
2.	Nostalgia proneness (NI)	0.65^***^	--					
3.	Horizontal collectivism	0.19^***^	0.23^***^	--				
4.	Vertical collectivism	0.23^***^	0.26^***^	0.54^***^	--			
5.	Horizontal individualism	0.09^*^	0.07^*^	0.31^***^	0.27^***^	--		
6.	Vertical individualism	0.05	0.12^***^	0.28^***^	0.24^***^	0.35^***^	--	
7.	Gender (0 = men, 1 = women)	0.04	0.06	0.10^**^	−0.05	−0.06	−0.17^***^	--
8.	Age	0.05	0.05	0.04	0.14^***^	0.10^**^	−0.10^**^	−0.07^*^

*N* = 868.

* *p* < 0.05;

** *p* < 0.01;

*** *p* < 0.001.

Consistent with prior findings (Singelis et al., 1995), the two horizontal dimensions (horizontal collectivism and horizontal individualism) and the two vertical dimensions (vertical collectivism and vertical individualism) were significantly and positively correlated. In addition, horizontal collectivism and vertical individualism were positively correlated, as were horizontal individualism and vertical collectivism. This overlap between the collectivism and individualism dimensions raises a legitimate question concerning their unique relations with nostalgia proneness. To address this, we first tested the partial correlation of each nostalgia-proneness measure with each collectivism dimension, controlling for the individualism dimensions. The positive associations between nostalgia-proneness measures and collectivism dimensions remained significant, when controlling for individualism dimensions. To be precise, the SNS was significantly correlated with horizontal collectivism (*pr* = 0.17, *p* < 0.001) and vertical collectivism (*pr* = 0.21, *p* < 0.001), and the NI was also significantly correlated with horizontal collectivism (*pr* = 0.21, *p* < 0.001) and vertical collectivism (*pr* = 0.24, *p* < 0.001). Next, we tested the partial correlation of each nostalgia-proneness measure with each individualism dimension, controlling for the collectivism dimensions. The SNS was not significantly associated with horizontal individualism (*pr* = 0.01, *p* = 0.751) or vertical individualism (*pr* = −0.02, *p* = 0.566), when controlling for collectivism dimensions. The NI was not significantly correlated with horizontal individualism (*pr* = −0.03, *p* = 0.470) or vertical individualism (*pr* = 0.04, *p* = 0.226) either.

Finally, we examined correlations with demographic variables. Neither gender nor age was significantly correlated with either measure of nostalgia proneness. Women evinced significantly higher vertical collectivism scores and lower vertical individualism scores than men. Older (compared to younger) individuals scored significantly higher on vertical collectivism and horizontal individualism and lower on vertical individualism. When we controlled for age and gender (in partial-correlation analyses), the correlations of both nostalgia measures with both collectivism dimensions were practically identical.

A key limitation of Study 1 concerns the exclusive focus on relational collectivism. This raises the question whether Study 1 findings can be replicated conceptually when collectivism is assessed in terms of one’s identification with more abstract, larger social entities. We tested this possibility in Study 2.

## Study 2

In Study 2, we examined the relation between nostalgia proneness and group collectivism. We assessed group collectivism in terms of participants’ identification with their national ingroup.

### Method

#### Participants

We tested 202 University of Thessaly students, who took part for extra course credit. We excluded four students because they were not Greek citizens, and six students because they did not complete all measures. The final sample comprised 192 individuals (188 women and 4 men), ranging in age from 18 to 40 years (*M* = 21.09, *SD* = 3.94).

#### Procedure and Materials

Participants responded to the materials individually, on a computer. As in Study 1, we assessed nostalgia proneness with the SNS (Barrett et al., 2010; α = 0.91, *M* = 5.04, *SD* = 1.13) and NI (Batcho, 1995; α = 0.87, *M* = 4.38, *SD* = 1.04). We assessed national identification with Leach et al. (2008) 14-item ingroup identification scale, which participants filled out in reference to their Greek nationality (1 = *strongly disagree*, 7 = *strongly agree*). The ingroup identification scale comprises two facets. (1) Group-level self-definition captures the degree to which individuals see themselves as similar to the ingroup prototype (i.e., self-stereotyping) and perceive ingroup members as similar to each other (i.e., in-group homogeneity). (2) Group-level self-investment encapsulates individuals’ positive feelings about their ingroup membership (i.e., satisfaction), the strength of their bond with the ingroup (i.e., solidarity), and the importance they ascribe to their ingroup memberships (i.e., centrality). We averaged across the relevant items to create indices of self-definition (e.g., “I have a lot in common with the average Greek person”; α = 0.89, *M* = 4.48, *SD* = 1.29) and self-investment (e.g., “I am glad to be Greek”; α = 0.89, *M* = 4.96, *SD* = 1.13).

### Results and Discussion

We present correlations between measured variables in Table 2. We did not include gender in the correlation matrix, because the sample included few men (*n* = 4). As hypothesized, both measures of nostalgia proneness were significantly and positively related to both group-level self-definition and group-level self-investment. Older participants scored lower on nostalgia (but age range was limited in this student sample). When we controlled for age (in partial correlation analyses), the correlations of SNS scores with self-definition (*pr* = 0.22, *p* = 0.003) and self-investment (*pr* = 0.20, *p* = 0.007) remained significant, as did the correlations of NI scores with self-definition (*pr* = 0.16, *p* = 0.028) and self-investment (*pr* = 0.26, *p* < 0.001).

**Table 2:** Correlations among nostalgia proneness, group-level self-definition, group-level investment, and age in Study 2.

S. No.	Measure	1	2	3	4
1.	Nostalgia proneness (SNS)	--			
2.	Nostalgia proneness (NI)	0.53^***^	--		
3.	Group-level self-definition	0.23^**^	0.17^*^	--	
4.	Group-level self-investment	0.21^**^	0.27^***^	0.60^***^	--
5.	Age	−0.17^*^	−0.12	−0.10	−0.10

*N* = 192.

* *p* < 0.05;

** *p* < 0.01;

*** *p* < 0.001.

Study 1 revealed that nostalgia proneness is associated with higher relational collectivism. Study 2 conceptually replicated and extended this finding by demonstrating that nostalgia proneness is also linked with higher group collectivism, as reflected in the strength of ingroup identification.

## General Discussion

### Summary of Findings

Extant evidence has tied nostalgia proneness to a stronger preference for engaging in activities with others (Batcho, 1998), increased empathy (Cheung et al., 2017a; Juhl et al., 2020; Newman et al., 2020), and greater emphasis on intimacy maintenance versus bitterness revival (Cheung et al., 2018). The present findings tighten further this nomological net around nostalgia proneness. Our point of departure was Brewer and Chen’s (2007) influential distinction between relational and group collectivism. Whereas relational collectivism involves self-definition in terms of one’s bonds to close others and small social networks, group collectivism denotes self-definition in terms of one’s membership in larger social groups or categories.

In Study 1, we examined the association of nostalgia proneness with relational collectivism (and individualism). This study further included a cross-cutting distinction between horizontal (i.e., emphasizing similarities between oneself and others) and vertical (i.e., emphasizing hierarchies and differences between oneself and others) orientations (Singelis et al., 1995; Triandis and Gelfand, 1998). Zero-order and partial correlations (controlling for individualism) revealed that high-nostalgia (compared to low-nostalgia) individuals scored higher on measures of horizontal and vertical collectivism. Zero-order correlations between nostalgia proneness and the individualism measures were small and rendered non-significant when we controlled for collectivism (in partial-correlation analyses). Study 2, in turn, demonstrated a link between nostalgia proneness and group collectivism, as reflected in two aspects of (national) ingroup identification: group level self-definition (i.e., the degree to which individuals see themselves as similar to the ingroup prototype and perceive ingroup members as similar to each other) and group level self-investment (i.e., the extent to which individuals value the ingroup and their membership in it). Across both studies, independent assessments of nostalgia proneness (SNS and NI) produced convergent support for the conclusion that high-nostalgia (compared to low-nostalgia) individuals are more likely both to emphasize the importance of maintaining tightly knit networks of interpersonal relationships, including the attendant rights and responsibilities (relational collectivism), and to value, identify with, and promote the collective welfare of more abstract, larger social groups (group collectivism). The magnitude of these relations (Study 1: *r* range = 0.19 to 0.26; Study 2: *r* range = 0.17 to 0.27) is typical for social and personality psychology research (Gignac and Szodorai, 2016; Funder and Ozer, 2019).

## Limitations and Future Directions

Our studies were correlational and, hence, did not allow us to examine direction of causality. However, prior research is consistent with the possibility of a bi-directional relation between collectivism and nostalgia proneness. Collectivism entails a strong need to belong (Bond and Smith, 1996; Suh, 2007) and there is now strong evidence that experimentally induced belongingness deficits, which heighten the need to belong (Hackenbracht and Gasper, 2013), lead to increased nostalgia (Zhou et al., 2008; Wildschut et al., 2010; Seehusen et al., 2013; Abakoumkin et al., 2017). Thus, collectivism may cause nostalgic reverie. Nostalgia, in turn, serves a compensatory role, assuaging the need to belong by strengthening social connectedness (for a review, see Sedikides and Wildschut, 2019). By so doing, nostalgia can increase the importance and strength of relational and group bonds. For example, discussing a shared nostalgic (vs. ordinary) memory with a close other (i.e., partner, friend, or family member) increases relationship positivity, intimacy, support, and mutual disclosure (Hepper et al., 2012b). Likewise, reflecting on nostalgic (vs. ordinary) events shared with other members of one’s university or national ingroup increases positive ingroup evaluations, charitable intentions toward the ingroup, and personal sacrifice for the ingroup (Wildschut et al., 2014), as well as ingroup bias in the evaluation of domestic and foreign consumer products (Dimitriadou et al., 2019). These findings point to a causal path from nostalgia to relational and group collectivism, particularly when one’s nostalgia pertains to, or is shared within, the collective in question. Explicating the bi-directional causal paths between nostalgia and (relational as well as group) collectivism presents a fruitful direction for research.

Another unresolved question is whether nostalgia proneness, through its positive association with group collectivism, is linked to increased prejudice and ethnocentrism. To understand the nuanced picture emerging from extant findings, two issues deserve careful consideration. First, it is important to distinguish between nostalgia for experiences from one’s personal past (i.e., the focus of our present studies) and nostalgia for the ostensibly glorious past of one’s group (e.g., national nostalgia). Proneness to nostalgia for one’s personal past is negatively correlated with prejudice against outgroups (Smeekes, 2015; Cheung et al., 2017a), and recalling a personally nostalgic (compared to ordinary) experience with a specific outgroup member reduces prejudice toward the entire outgroup (Turner et al., 2012, 2013, 2018). By contrast, nostalgia for the past of one’s group (e.g., “How often do you long for the good old days of the country?”) has been linked with higher prejudice, national glorification, and anger toward outgroups (Smeekes, 2015; Smeekes et al., 2015, 2018; Cheung et al., 2017b; Baldwin et al., 2018). Yet, findings by Martinovic et al. (2017) raise a second issue that requires attention, pertaining to the referent of nostalgia. Their findings indicate that, under certain circumstances, nostalgia for the collective past is associated with improved outgroup attitudes. Participants from former Yugoslavia rated their nostalgia for the Yugoslavia of the past (e.g., “I get nostalgic when I think back to Yugoslavia in the past times”) and then indicated their level of contact with members from different ethnic subgroups from former Yugoslavia (Bosniaks, Serbs, and Croats). Nostalgia for the superordinate group, Yugoslavia, predicted increased contact between ethnic subgroups. Furthermore, political orientation might affect the referent of nostalgia. For example, political conservatives might be more prone than political liberals to nostalgize about the “good old days of a country” and thereby identify with this country. Clarifying the precise circumstances under which nostalgia proneness, in its different guises, is either positively or negatively associated with outgroup prejudice presents a challenge for future research.

## Coda

High-nostalgia (compared to low-nostalgia) individuals attach great value and significance to establishing, protecting, and enhancing close-knit interpersonal relationships, as well as to maintaining the integrity, and promoting the interests, of more abstract, larger groups and social categories. As history indicates, these pursuits can be the source of both great happiness and intense suffering. The present findings mark a step along the path to understanding these profound and sometimes contradictory implications of nostalgia proneness and human sociality.

## Data Availability Statement

The datasets presented in Study 1 can be found in online repositories. The names of the repository/repositories and accession number(s) can be found at: www.surveydata.nl/liss-panel-data-archive. The datasets presented in Study 2 are available on request to the corresponding author.

## Ethics Statement

Study 1, which involved human participants, was reviewed and approved by University of Southampton, School of Psychology ethics committee. The treatment of Study’s 2 human participants complies with APA ethical standards and with the University of Thessaly Code of Conduct. The patients/participants in both studies provided their written informed consent to participate in these studies.

## Author Contributions

GA, TW, and CS conceptualized the studies (theory, hypotheses, and design). TW conducted Study 1 and GA conducted Study 2. GA and TW wrote drafts of the manuscript. CS provided feedback and revised the drafts. All authors contributed to the article and approved the submitted version.

### Conflict of Interest

The authors declare that the research was conducted in the absence of any commercial or financial relationships that could be construed as a potential conflict of interest.

## Footnotes

^1^www.surveydata.nl/liss-panel-data-archive
